# Vestibular Evoked Myogenic Potential in Vestibular Migraine: A Systematic Review of Diagnostic Utility

**DOI:** 10.3390/audiolres16010011

**Published:** 2026-01-17

**Authors:** Mayur Bhat, Krithi Rao, Sinchana Hegde, Kaushlendra Kumar, Aditya Khandagale, KM Prajwal, Shezeen Abdul Gafoor

**Affiliations:** Department of Audiology and Speech Language Pathology, Kasturba Medical College Mangalore, Manipal Academy of Higher Education, Manipal 576104, India

**Keywords:** vestibular migraine, vestibular evoked myogenic potential (VEMP), cervical vestibular evoked myogenic potential (cVEMP), ocular vestibular evoked myogenic potential (oVEMP), Migraine-associated Vertigo

## Abstract

**Background/Objectives:** Vestibular migraine (VM) is one of the most prevalent causes of episodic vertigo, yet it remains underdiagnosed due to overlapping features with other vestibular disorders and the absence of definitive diagnostic tests. Vestibular evoked myogenic potentials (VEMPs) assess otolith and vestibular nerve function and may help identify pathophysiological mechanisms in VM. This systematic review aimed to evaluate the usefulness of VEMP in understanding VM, synthesize existing findings, and explore its clinical implications. **Method:** A systematic search was performed in PubMed, ProQuest, Scopus, Web of Science, and EMBASE up to 2025 following PRISMA guidelines. Studies were included if they assessed cVEMP and/or oVEMP in patients diagnosed with VM using established clinical criteria. Data extraction and quality assessment were conducted independently by three reviewers using Cochrane and Joanna Briggs Institute tools. A total of 2578 titles and abstracts were screened, and 28 studies met the inclusion criteria. **Results:** Across 28 studies, 23 reported VEMP abnormalities in VM. The most frequent findings were reduced amplitudes and increased asymmetry ratios compared to healthy controls, indicating potential otolithic dysfunction. Latency prolongations were less consistently reported. Differences between cVEMP and oVEMP findings in individuals with VM suggested variable involvement of saccular and utricular pathways, with oVEMP abnormalities appearing more prominent. **Conclusions:** VEMP testing reveals subtle vestibular dysfunction in VM, primarily reflected in reduced amplitude and altered asymmetry ratios. However, the association between VEMP abnormality and VM is inconclusive, specifically due to heterogeneity among the included studies. Although findings support its potential as a diagnostic adjunct, methodological variability (including variability in patient recruitment) underscores the need for standardized VEMP protocols to enhance diagnostic accuracy and comparability across studies.

## 1. Introduction

Vestibular migraine (VM) is a clinical condition marked by recurring vestibular symptoms, including vertigo, dizziness, and imbalance, which frequently occur alongside migraine features such as headache, sensitivity to light (photophobia), and sound (phonophobia). Vertigo episodes may last from a few seconds to several days and can happen spontaneously or be triggered by changes in position [1]. 

VM affects an estimated 1% to 3.2% of adults [2], making it the most common cause of episodic vertigo in this population. It is more prevalent among women [3] and individuals with a personal or family history of migraines [4]. Vestibular disorders affect between 0.4% and 15% of children [5]. VM is among the leading causes of vertigo in this age group, responsible for 24% of all cases [5,6]. Among children aged 3 to 17 years, the prevalence of vestibular symptoms is estimated to range from 5.3% to 8% [7].

The pathophysiology of VM involves a complex interplay of central and peripheral vestibular dysfunction, trigemino-vascular system activation, brainstem and cortical involvement, genetic and environmental factors, and possibly localized neuroinflammation [8]. 

Differential diagnosis in VM is essential because of its heterogeneous clinical presentation and the lack of specific clinical signs or objective diagnostic measurements [9]. Major challenges include variability in dizziness characteristics, attack duration, and the timing of headache or other migrainous features relative to vestibular symptoms. Accurate differential diagnosis is necessary to distinguish VM from other causes of dizziness and to guide appropriate management. VM is frequently misdiagnosed as Ménière’s disease or benign paroxysmal positional vertigo (BPPV) due to overlapping symptoms [10].

The vestibular evoked myogenic potential (VEMP) assesses vestibular system function, particularly the otolith organs responsible for detecting linear acceleration and head position relative to gravity. VEMP responses are elicited by sound, vibration, or electrical stimulation and recorded from specific muscles. The most commonly used types are cervical VEMP (cVEMP), recorded from the sternocleidomastoid muscle to evaluate saccule and inferior vestibular nerve function, and ocular VEMP (oVEMP), recorded from the inferior oblique muscle to assess utricle and superior vestibular nerve function [11].

Despite its high prevalence, VM remains underdiagnosed due to overlapping symptoms with other vestibular disorders and the absence of a definitive diagnostic test [12]. Several studies have reported abnormal VEMP responses in VM patients, suggesting dysfunction in the otolith organs or central vestibular processing [13]. The amplitude of the P1-N1 wave in VEMP testing was found to be significantly different in patients with VM compared with controls, indicating asymmetric effects on neuroanatomical structures in the lower brainstem [14]. However, conflicting findings exist, with some studies reporting normal VEMP responses in VM patients, complicating its role as a definitive diagnostic biomarker [15]. Given the growing interest in utilizing VEMP as a potential diagnostic and pathophysiological marker for VM, a systematic review is warranted to synthesize the available evidence. This review aims to evaluate the role of VEMP in VM, assess the consistency of findings across studies, and explore its clinical implications for diagnosing and understanding vestibular dysfunction in migraine patients. By consolidating current knowledge, this review will help determine whether VEMP can serve as a reliable diagnostic tool and guide future research in the field.

## 2. Materials and Methods

This systematic review was conducted in accordance with the Preferred Reporting Items for Systematic Reviews and Meta-Analyses (PRISMA) guidelines [16]. The review aimed to assess the role of vestibular evoked myogenic potentials (VEMPs) in the diagnosis and characterization of vestibular migraine (VM).

### 2.1. Keyword Build

Keywords for the search strategy were built using the Medical Term Search engine by Cochrane [17] and with inputs from experienced researchers working in the field of auditory and vestibular diagnosis and management. The keywords related to "Vestibular Evoked Myogenic Potential” and “Vestibular Migraine” were identified and used in combination with appropriate Booleans to obtain articles of interest. The final list of keywords is provided below:

- Vestibular Evoked Myogenic Potential;
- VEMP;
- Vestibular Migraine;
- Migraine-associated Vertigo;
- Vestibular Dysfunction.

The search strategy used was—(((Vestibular Migraine[Title/Abstract]) OR (Migraine-associated Vertigo[Title/Abstract])) OR (Vestibular Dysfunction[Title/Abstract])) AND ((Vestibular Evoked Myogenic Potential[Title/Abstract]) OR (VEMP[Title/Abstract])).

### 2.2. Search Strategy

These keywords were used to search five major databases, including PubMed, ProQuest, Scopus, Web of Science, and EMBASE (performed on 10 April 2025). The search was predominantly run for the title and/or abstract for all articles published till April 2025. The reference lists of relevant articles were also screened for additional studies. A total of 3840 articles were imported from the search engine into Rayyan software (https://www.rayyan.ai/) [18], out of which 1262 duplicates were detected. After removing duplicates, a total of 2578 articles were retained for title and abstract screening.

### 2.3. Screening (Title, Abstract, and Full-Length)

The first and second authors scanned 2578 articles from the web search engine for inclusion using the selection criteria. The PRISMA flow chart contains the specifics of the screening. The third author settled any disagreement between the first and second author’s decisions. “Rayyan,” an online systematic review program suggested by Cochrane, was used for title and abstract screening, full-text screening, and data extraction.

Articles were included for systematic review if they met the following criteria:

- Published in peer-reviewed journals.
- Involved human participants diagnosed with vestibular migraine based on established clinical criteria (e.g., Bárány Society criteria or International Classification of Headache Disorders [ICHD]).
- Assessed VEMP responses (cVEMP, oVEMP, or both) in VM patients.
- Reported quantitative VEMP findings, including amplitude, latency, or threshold measurements.
- Available in English.

Studies were excluded if they met the following criteria:

- Focused on animal models or in vitro studies.
- Lacked original research (e.g., reviews, meta-analyses, commentaries, or editorials).
- Did not provide sufficient data on VEMP parameters in VM patients.

After full-text screening, 2485 articles were excluded from the review due to inappropriate study design (1096), non-relevant population (842), or unsuitable outcome measures (547). A total of 77 articles were included for full-text screening, of which 28 articles were selected for evidence synthesis.

### 2.4. Data Extraction

A data extraction form was created based on the standard template given by Cochrane. Two authors independently extracted data from each article, and the third author checked each form to validate the extracted data. General information, such as study samples, their age, and gender, was extracted from each study. Necessary information like hearing status, handedness, stimulus characteristics, and transducer, as well as equipment-related information, such as design, manufacturers, and channel specification, was also extracted. The primary outcome measures included the following:

- Latency and amplitude of P1 and N1 components.
- Any other quantitative assessments, like PTA, VNG, or caloric tests.

### 2.5. Study Quality Assessment

Risk factor studies were assessed using the non-randomized version of the Mixed Methods Appraisal tool (MMAT V 2018) [19]. Three audiology-experienced professional reviewers evaluated the listed studies’ quality. The quality evaluations of the included studies were carried out by two reviewers. The third reviewer’s score was crucial if the first two reviewers could not agree.

## 3. Results

### 3.1. The Study Selection Process

**Figure 1:**
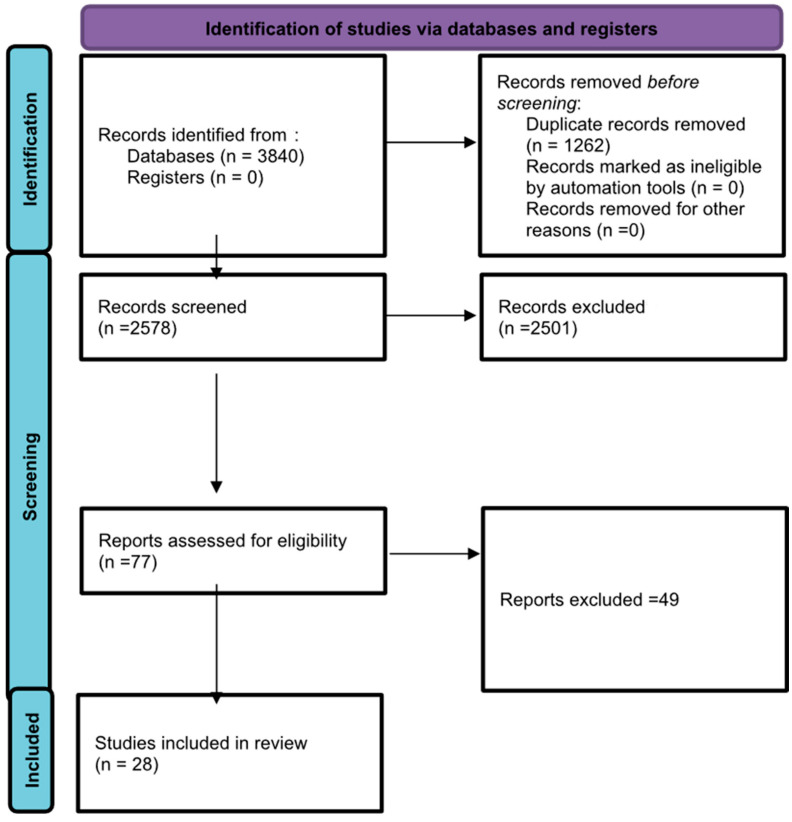
PRISMA flowchart explaining the screening and selection of the studies.

The PRISMA flowchart in Figure 1 illustrates the overall results and the process involved in selecting and rejecting studies that were part of the review process. The initial searches across the five included databases yielded 3840 scientific papers about vestibular migraine (VM) and vestibular evoked myogenic potentials (VEMPs). After removing duplicate entries, 2578 titles and abstracts were screened. Out of these, 77 articles were read in full and checked carefully. Ultimately, 28 studies met all the requirements and were selected for the final review. The findings of the risk of bias analysis are presented in Table 1.

### 3.2. General Characteristics

#### 3.2.1. Study Design and Population

Among the 28 selected studies, researchers employed different methods: 12 studies were case–control, 13 were cross-sectional, 2 were prospective cohort studies, and 1 was a pre- and post-treatment study design. These studies examined a total of 1898 subjects, divided into different groups based on inclusion or exclusion criteria. The included studies involved 557 healthy controls and 895 participants with VM, of whom 5 had mild to moderate symmetrical sensorineural hearing loss, 96 had ordinary migraine, 47 had migraine without aura, and 303 had Ménière’s disease. Not all of these groups are included in every study, though. There were eight studies with a VM and a control group; six with VM, MD, and control groups; five with just a VM group; four with VM, migraine, and control groups; one with migraine and VM; one with VM and MD; and one with VM, MD, migraine, and control groups.

#### 3.2.2. Demographics and Clinical Profile

Participants’ mean age was 40.51 years, and the standard deviation was 6.81, where the mean of the VM group was 40.23 years and the SD was 5.68, the mean of the MD group was 47.92 years and the SD was 8.17, the mean of the migraine group was 35.81 years and the SD was 2.99, the mean of the migraine without aura group was 34.1 years and the SD was 4.10, and the mean of the control was 38.33 years and the SD was 5.41. The male-to-female ratio in the majority of the study was not equal, as more females were recruited than males. The total number of females was 1058, and the number of males was 356. In the VM group, the total number of females was 603, and the number of males was 161. Again, this reflects the general trend of more women experiencing migraines. However, the actual reporting of gender breakdown was not always consistent across the studies, with three studies not providing this information

Pure-tone audiometry was used to check the hearing status, and hearing sensitivity was considered “normal” if the pure tone average was ≤15 dB HL. The hearing status of VM subjects involved in the investigations varied: only one study included five individuals with mild to severe symmetrical sensorineural hearing loss, with normal-hearing VM participants [26], whereas 27 studies included individuals with normal hearing. Details on overlapping hearing symptoms were rare, though symptoms like tinnitus were often noticed in people with VM.

### 3.3. Assessment Method

#### 3.3.1. Primary Outcome—Vestibular Evoked Myogenic Potential (VEMP)

All included studies evaluated the vestibular function of participants using cervical (cVEMP) and/or ocular VEMP (oVEMP), which assess the saccular and utricular pathways, respectively. Testing was primarily conducted via air-conducted tone bursts or click stimuli, with one study using tone pips [26]. Across all studies, the stimuli were delivered through inserts or headphones, except one study, which employed speakers [35]. Stimulus frequencies ranged from 400 Hz to 2000 Hz, with 500 Hz tone bursts being the most commonly used and considered the most reliable. Stimulus intensity varied between 90 and 130 dB SPL, and the number of sweeps ranged from 100 to 200. Five studies used frequency tuning or multifrequency VEMP in an attempt to differentiate between VM and MD [31,33,43,44]. Rarefaction polarity was most frequently employed, although alternating polarity was used in three studies [31,34,41] and condensation in one [36]. The rise–plateau–fall durations varied across studies, including patterns such as 2–2–2 ms [20,21], 2–0–2 ms [25,39], 2.5–2.5–2.5 ms [14], and 1–2–1 ms [13,24,30]. The repetition rate of stimuli ranged from 3/s to 9/s, with 5.1/s being the most commonly applied. Electrode placements were largely similar across studies (as summarized in Table 2), with minor variations. It was not feasible to provide a single standard set of values that would apply to all studies because of these variations in stimulus parameters and recording protocols.

#### 3.3.2. Secondary Outcome—Other Vestibular and Auditory Tests

In addition to VEMP, 16 studies employed supplementary assessments to evaluate auditory and vestibular function. These included pure tone audiometry (PTA), auditory brainstem response (ABR), caloric testing, video head impulse testing (vHIT), videonystagmography (VNG), Fukuda testing, and tandem gait testing. However, not all studies used every test. One study administered the motion sickness susceptibility questionnaire [28], two studies included the Dizziness Handicap Index (DHI) [30,34], and one study included the Tinnitus Handicap Inventory (THI) and Hospital Anxiety and Depression Scale (HADS) questionnaires [30]. These tests were primarily used to provide a broader evaluation of vestibular and auditory performance alongside VEMP.

### 3.4. Outcome Measures

The outcome measures included were the latency and peak-to-peak amplitude of P1 and N1 peaks in cVEMP and N1 and P1 in oVEMP. Also, the asymmetrical ratio between both sides was considered. One study considered the 500–1000 Hz VEMP slope [33], and two studies correlated VEMP results with the severity of vestibular complaints using standardized questionnaires like the dizziness handicap index and motion sickness questionnaires, giving a more complete view of how VM affects daily functioning [28,30].

### 3.5. Extraction Results

A total of 23 studies reported that people with VM had abnormal VEMP responses (Table 3). The most common finding was that VM participants had reduced VEMP amplitude, prolonged latency, or absent VEMP when compared to the control group. A total of five studies showed there was no statistical significance between VM and other groups in VEMP parameters [15,25,27,31,32].

#### 3.5.1. Primary Outcomes

Across 28 studies investigating VEMP findings in VM, notable variability was observed in latency, amplitude, and response characteristics when compared to control groups (CGs) and other vestibular disorders, such as Ménière’s disease (MD). Four studies reported prolonged latencies of the P and N peaks in both cVEMP and oVEMP of VM participants relative to healthy controls [33,37,39,41], and one study found the latency prolongation only in cVEMP and not in oVEMP [36], while another demonstrated prolonged latency in MD compared to VM, suggesting possible disorder-specific differences in neural conduction timing [29].

Amplitude abnormalities were the most frequently reported, wherein thirteen studies showed reduced VEMP amplitudes in VM compared to controls. Of these, one study noted a decrease limited to the P-wave amplitude rather than the peak-to-peak amplitude [24]. While one study observed a greater amplitude reduction in oVEMP than in cVEMP, indicating stronger utricular pathway involvement [21], another study reported amplitude reduction exclusively in oVEMP but not in cVEMP [29]. One study stated that the amplitude differences are clear during migraine attacks and tend to be less evident in the periods between attacks, hinting at a fluctuating nature of balance issues in VM [22].

The asymmetry ratio (AR) in VEMP is a measure of the difference in response amplitude between the left and the right ears, calculated by comparing the VEMP amplitudes of the two sides, and is calculated as

AR = 100’ (larger VEMP − smaller VEMP)/(larger VEMP + smaller VEMP).

The AR differences were reported in two studies. One of these found a more marked AR difference in cVEMP than in oVEMP, suggesting a predominant saccular or inferior vestibular nerve involvement [34]. Additionally, when comparing VM and MD, one study found that both amplitude reduction and AR were greater in MD [23], whereas another reported a higher amplitude ratio in VM [25].

Four studies documented absent VEMP responses in VM participants. Furthermore, one study found that responses were more frequently absent when stimuli of 100 dBnHL were used compared to 90 dBnHL, indicating a possible sensitivity to stimulus intensity [26]. Another study comparing pre- and post-intervention VEMP responses, where Flunarizine (10 mg) was given once daily for two consecutive months, observed no significant difference between the pre- and post- measurement [32]. One study also noted a higher incidence of abnormal oVEMP than cVEMP responses in VM [38].

Other VEMP parameters provided further diagnostic insight. Frequency-tuning characteristics differentiated MD from VM in one study, where MD had lower response rates at 500 Hz compared to 1000 Hz, supporting their value in distinguishing between these disorders [20]. Another study reported a decreased 500–1000 Hz slope in VM [33]. Prognostic factors were also examined; one study observed that patients with bilateral oVEMP responses and a cVEMP asymmetry ratio below 40% tended to have a more favorable clinical outcome [30].

Overall, most studies indicate that individuals with VM exhibit subtle but consistent abnormalities in VEMP responses, most prominently reflected in reduced amplitudes and increased ARs compared to healthy controls. Latency prolongation was less consistently observed. Differences between cVEMP and oVEMP findings suggest that both saccular and utricular pathways may be variably affected in VM, with some evidence of greater cVEMP asymmetry and oVEMP amplitude reduction.

#### 3.5.2. Secondary Outcomes

In addition to VEMP assessments, several studies evaluated supplementary vestibular and auditory measures to provide a broader understanding of vestibular function in vestibular migraine (VM). One study reported that caloric test abnormalities were more frequent than vHIT deficits [21]. Another study found that VM participants often exhibited normal vHIT and cVEMP findings but demonstrated abnormal gaze stabilization on the functional head impulse test (fHIT), indicating possible central or visual–vestibular integration deficits [23].

Motion sickness susceptibility was consistently higher in VM compared to migraine without vestibular symptoms, reflecting heightened vestibular sensitivity in this population [28]. Another study also reported abnormal videonystagmography (VNG) findings in 94% of VM subjects, suggesting a wider spectrum of vestibular dysfunction [38].

Correlations between electrophysiological and postural measures were explored in another study, where prolonged VEMP latencies were associated with impaired performance on the Fukuda stepping test, tandem gait, and the modified Clinical Test of Sensory Interaction on Balance, indicating that delayed vestibular responses may manifest as measurable balance instability [39]. Additionally, one study reported abnormal bithermal caloric responses [41], and another showed pathological nystagmus in VM [42]. Auditory brainstem response (ABR) testing in another study revealed a prolonged fifth wave latency (>6 ms), suggesting delayed neural transmission along the auditory brainstem pathways [22].

One study showed that 3D-real-IR MRI helps differentiate VM and MD. VM and MD behaved similarly in vestibular dysfunction and their transduction pathways, but MD appeared to be more severe than VM [35].

## 4. Discussion

VM is a prevalent neurological disorder characterized by episodic vertigo and other vestibular symptoms in individuals with a history of migraine, posing significant diagnostic challenges due to its variable clinical presentation [37]. VEMP is a test that can reveal abnormalities in both peripheral and central vestibular pathways in VM patients [37]. However, findings across studies are conflicting, as some studies report no differences in VEMP findings between controls and individuals with VM [8], while others report absent VEMP responses in VM [28]. Hence, this study aimed to systematically review the literature to explore the utility of VEMP in VM. The initial searches across five databases found 3840 scientific papers related to VM and VEMPs. After removing duplicate entries, 2578 titles and abstracts were screened. Out of these, 77 articles were read in full and checked carefully. Ultimately, 28 studies met all the requirements and were selected for the final review.

### 4.1. General Characteristics of the Studies

A majority of studies compared VEMP findings between normal-hearing subjects and those with VM. Other studies used clinical populations like migraine and MD to compare VEMP abnormalities. Several studies reported a significant overlap between MD and VM. The occurrence of migraine is more common in MD when compared to the general population [45]. Additionally, VM patients may experience symptoms such as tinnitus and aural fullness [46]. Such overlap between these different vestibular pathologies demands greater caution when establishing a final diagnosis. Currently, the specific manifestations of VM in isolation are unclear, which complicates matters further when it overlaps with other conditions. This difficulty is reflected in the fact that the existing research findings are mixed. In the current study, although the evidence is weak (as a result of heterogeneity amongst studies), it seems as though VEMP abnormalities are higher in MD than VM, and differential diagnosis is better achieved when utilizing a test battery approach.

Our results also showed that women were more likely than men to have VM, which is consistent with research showing that women are 1.5–5 times more likely than men to have VM [3]. It has been suggested that VM has a genetic etiology, specifically an autosomal dominant pattern of inheritance with lower penetrance in men, although the exact causes are unknown [47]. Furthermore, our findings also revealed hearing loss as an uncommon symptom, as only one study has reported and recruited VM subjects with hearing loss. Auditory manifestation in VM is poorly understood, as one study reports hearing loss in only 9% of individuals with VM [3]. One study reported a higher incidence of high-frequency hearing loss when compared to loss at standard audiometric frequency, stressing the importance of extended high-frequency audiometry in VM evaluation [48].

### 4.2. VEMP Protocols Used Across Studies

The exact protocol for robust VEMP recordings could not be deduced in the current study, as there was variability in the protocol followed, stressing the importance of developing universal standards for VEMP evaluations. Nonetheless, we may discern some essential characteristics crucial for documenting robust VEMP. A large majority of studies used short-duration stimuli like clicks and tone bursts to record VEMP. Because clicks have a quick onset and stimulate across a range of frequencies, clicks (0.1 ms square waves) provide a good estimate of the VEMPs. Similarly, low-frequency tone bursts are also effective stimuli for recording VEMPs because transmission to the saccule demonstrates frequency tuning, with the preferred frequency at roughly 500–1000 Hz [49]. In line with this, the majority of the included studies (18/28) utilized 500 Hz tone-bursts to record VEMPs. The results of a few recent investigations that used the frequency-tuning approach, which recorded VEMP at several frequencies, showed that MD participants had greater frequency tuning abnormalities than VM subjects [43,44]. Though the results are promising, additional exploratory research of this kind is required to confirm the usefulness of frequency-tuning VEMPs in the differential diagnosis of MD and VM. The majority of the studies used high-intensity stimuli ranging from 90 dB to 130 dB SPL. Unlike the cochlea, the vestibular system is normally shielded from environmental sound, and only very loud sounds are sufficiently intense to activate the otolith hair cells [11]. Regarding repetition rate, lower repetition rates are optimal because they produce stronger, more consistent, and more efficient VEMP responses, while higher rates can lead to reduced amplitude and longer latencies [11]. In line with this, most included studies used lower repetition rates, ranging from 5.1 to 11.1/s.

### 4.3. VEMPs in VM

The extracted results revealed a significant VEMP abnormality in 23 out of 28 articles, indicating the diagnostic importance of this test in VM evaluations. Only five studies showed no significant difference in VEMP findings between VM and the other group. Though a majority of studies reported VEMP abnormalities, there was no consistency in measuring and reporting outcome measures. Only three studies reported prolongation in latencies of the P and N components, indicating that latency abnormalities may not be significant in VM subjects.

On the other hand, the majority of studies consistently reported a reduction in peak-to-peak amplitudes of P and N components when compared to controls. The prolongation of latency and decrease in amplitude could be attributed to damage to the saccule, utricle, or central vestibular pathway, or their innervating structures, in individuals with VM [37]. VM is complex and may involve both central and peripheral vestibular systems. While prolonged latency points to central issues, reduced amplitude might indicate peripheral dysfunction, leading to mixed results depending on the primary involvement in an individual patient [39]. The number of studies that measured AR in individuals with VM is scant. However, the few studies that measured it reported abnormal AR in this population [23,34,40].

Regarding differential diagnosis, studies that compared VEMP findings in individuals with MD vs. VM [13,20,23,29,31,33,35,40] reported prolonged latency and reduced amplitude in MD when compared to individuals with VM. One possible reason for this could be attributed to the site of impairment. While VM is most commonly believed to be a central nervous system condition associated with the migraine pathway, where the peripheral structures may be less affected, MD is a peripheral disorder that primarily affects inner ear structures [50].

The current review findings also extracted differences reported in cVEMP and oVEMP and found significant variabilities in the study findings. While some studies have reported abnormalities in cVEMP, others have reported abnormal oVEMPs. cVEMP and oVEMP, which assess sacculocollic and utriculo-ocular pathways, respectively, provide valuable insights into otolithic dysfunction in VM patients [37]. Specifically, these findings support the hypothesis that VM involves both the saccular and utricular pathways, contrary to the notion that VM is a purely central pathology. However, the specific patterns and diagnostic utility of cVEMP and oVEMP abnormalities in VM are still unclear, with several studies reporting conflicting findings regarding latency and amplitude parameters [31,37]. The utriculo-ocular reflex, which is assessed by oVEMP, appears to be particularly affected, suggesting a potential biomarker for VM [51]. This is further supported by the finding that VM patients are more likely to exhibit normal cVEMP responses in the presence of unilaterally abnormal oVEMP responses, highlighting a distinct pattern that may aid in the diagnosis of VM [51]. Taken together, the current review reveals that VEMP can enhance the diagnostic workflow for VM by providing objective evidence of otolith pathway involvement and helping differentiate VM from other vestibular disorders, especially MD.

## 5. Limitations and Future Direction

Though a majority of studies have shown significant VEMP abnormalities in VM subjects, results show significant variability in reporting outcome measures of VEMP. The differences in findings could be due to patient selection criteria, the difference in diagnostic criteria used for VM, other stimuli, and recording parameters used for the recording of VEMP. Also, a few studies have shown a high risk of bias in subject allocation and sampling, and hence, the interpretation of the present study’s results requires careful consideration. Future studies should focus on systematically categorizing the studies based on the variabilities and quantifying the impact of these variabilities on the pooled data.

Combining VEMP with other vestibular tests, such as the video head impulse test (V-HIT), posturography, and caloric testing, may improve diagnostic accuracy and help differentiate VM from other vestibular disorders. Hence, the current study stresses the importance of developing standardized protocols that integrate multiple tests (including VEMPs) to obtain a more comprehensive assessment.

## 6. Summary and Conclusions

The current study aimed to evaluate the role of VEMP in VM, assess the consistency of findings across studies, and explore its clinical implications for diagnosing and understanding vestibular dysfunction in VM patients. The initial searches of the five databases found 3840 scientific papers on vestibular migraine (VM) and vestibular evoked myogenic potentials (VEMPs). Out of these, 77 articles were read in full, and 28 studies fit all the requirements and were chosen for the final review. Overall, most studies indicate that individuals with VM exhibit subtle abnormalities in VEMP responses, most prominently reflected in reduced amplitudes and increased asymmetry ratios compared to healthy controls. Latency prolongation was less consistently observed. Differences between cVEMP and oVEMP findings suggest that both saccular and utricular pathways may be variably affected in VM, with some evidence of greater oVEMP abnormalities suggesting a potential biomarker for VM.

## Figures and Tables

**Table 1:** Results of risk of bias assessment. The tick marks indicate that there is no bias, whereas the cross indicates that there is some possible bias or unclear information.

Studies	Are There Clear Research Questions?	Do the Collected Data Help Address the Research Questions?	Is the Sampling Strategy Relevant to Addressing the Research Question?	Is the Sample Representative of the Target Population?	Are the Measurements Appropriate?	Is the Risk of Nonresponse Bias Low?	Is the Statistical Analysis Appropriate to Answer the Research Question?
Salviz et al. 2015 [20]	✓	✓	✓	✓	✓	✓	✓
Özdemir et al., 2019 [15]	✓	✓	✓	✓	✓	✕	✓
Fu et al., 2021 [21]	✓	✓	✓	✓	✓	✕	✓
Sürmeli et al., 2019 [22]	✓	✓	✓	✓	✓	✓	✓
Balayeva et al., 2013 [23]	✓	✓	✓	✓	✓	✓	✓
Yetiser et al., 2016 [24]	✓	✓	✓	✓	✓	✕	✓
Baier et al., 2009 [14]	✓	✓	✓	✓	✓	✓	✓
Moallemi et al., 2015 [25]	✓	✓	✓	✓	✓	✓	✓
Hong et al., 2011 [26]	✓	✓	✓	✓	✓	✕	✓
Kandemir et al., 2013 [27]	✓	✓	✓	✓	✓	✓	✓
Boldingh et al., 2011 [28]	✓	✓	✓	✓	✓	✕	✓
Rizk et al., 2020 [29]	✓	✓	✓	✓	✓	✓	✓
Goto et al., 2024 [30]	✓	✓	✕	✕	✓	✕	✓
Zuniga et al., 2012 [13]	✓	✓	✓	✓	✓	✕	✓
Taylor et al., 2012 [31]	✓	✓	✓	✓	✓	✕	✓
Islam et al., 2023 [32]	✓	✓	✕	✕	✓	✕	✓
Murofushi et al., 2009 [33]	✓	✓	✓	✓	✓	✓	✓
Dispenza et al., 2021 [34]	✓	✓	✕	✕	✓	✓	✓
Sun et al., 2017 [35]	✓	✓	✓	✓	✓	✓	✓
Elmoazen et al., 2020 [36]	✓	✓	✓	✓	✓	✓	✓
Sanitha and Sinha et al., 2024 [37]	✓	✓	✓	✓	✓	✓	✓
Swain et al., 2020 [38]	✓	✓	✕	✕	✓	✓	✓
Khalil et al., 2016 [39]	✓	✓	✓	✓	✓	✓	✓
Utkur et al., 2013 [40]	✓	✓	✓	✓	✓	✓	✓
Sanyelbhaa Talaat and Sanyelbhaa Talaat, 2014 [41]	✓	✓	✓	✓	✓	✓	✓
Nafie et el., 2011 [42]	✓	✓	✕	✕	✓	✓	✓
Tang et al., 2025 [43]	✓	✓	✓	✓	✓	✓	✓
Anand and Sarda, 2025 [44]	✓	✓	✓	✓	✓	✓	✓

**Table 2:** Electrode montage used for recording VEMP.

Electrode	cVEMP (Ipsilateral Recording)	oVEMP (Contralateral Recording)
Ground	Forehead	Forehead
Non-inverting	Middle of the sternocleidomastoid (SCM) muscle	1 cm below the lower eye lid
Inverting	Upper sternum	2 cm below the non-inverting

**Table 3:** Results of data extraction for all 28 studies included in the review.

Study	Sample	Stimulus and Potential Recorded	Outcome Measures	Key VEMP Findings
Salviz et al., 2016 [20]	22 VM, 18 controls	AC TB 500/1000 Hz, cVEMP on SCM	Latency, amplitude, FR, AR	VM showed reduced 500-Hz amplitudes; response rates were similar to controls.
Özdemir et al., 2019 [15]	31 VM, 32 migraine	500 Hz TB cVEMP on SCM	Latency, amplitude, AR	No significant differences between migraine and VM.
Fu et al., 2021 [21]	41 VM (pre- and post-treatment)	500 Hz TB; cVEMP and oVEMP	Presence of response, amplitude, AR vHIT, VNG, caloric test	20% abnormal cVEMP, 44% abnormal oVEMP; some abnormalities in other vestibular tests.
Sürmeli et al., 2016 [22]	32 VM, 27 migraine, 27 controls	AC Clicks cVEMP	Amplitude and latency, ABR	Reduced cVEMP amplitude in VM; latencies were normal.
Balayeva et al., 2023 [23]	22 VM, 21 MD, 21 controls	AC Clicks cVEMP	Amplitude, AR, vHIT, fHIT, PTA	VM normal cVEMP; MD showed significantly reduced amplitudes.
Yetiser et al., 2016 [24]	30 females migraine, 15 age-matched controls	500 Hz TB (AC) cVEMP	Latency, amplitude, AR	No difference in latency. Reduced P1 amplitudes in patients. 13.4% showed high AR (left ear predominance in pathology).
Baier et al., 2009 [14]	63 VM, 63 controls	400 Hz TB cVEMP on SCM	Latency, amplitude, SVV, caloric test	VM showed significantly lower amplitudes; latencies were normal.
Moallemi et al., 2015 [25]	25 VM, 26 controls	500 Hz TB cVEMP on SCM	Latency, amplitude, AR	No significant group differences.
Hong et al., 2011 [26]	30 VM, 31 controls	500 Hz tone pips (90 and 100 dB nHL)	Latency, amplitude, incidence, AR	More absent responses in VM. AR and latencies similar.
Kandemir et al., 2013 [27]	24 VM, 20 migraine without aura, 20 tension-type headache, 30 CG	AC Clicks (100 dB nHL) cVEMP on SCM	Latency, AR, caloric test	Latencies normal across groups; no diagnostic differences.
Boldingh et al., 2011 [28]	37 VM, 32 migraine, 30 controls	Clicks	Presence, threshold, latency	Absent responses more common in VM (44%) vs. controls (3%).
Rizk et al., 2020 [29]	34 VM, 25 MD, 13 controls	500 Hz TB (cVEMP and oVEMP)	Latency, amplitude, AR	cVEMP: no differences; oVEMP: reduced amplitudes and earlier latency in VM vs. controls.
Goto et al., 2024 [30]	25 VM pre–post treatment	500 Hz TB (cVEMP and oVEMP)	Presence, amplitude, AR, posturography, vHIT, DHI	High rate of absent oVEMP; VEMP abnormalities predicted poorer prognosis.
Zuniga et al., 2012 [13]	21 VM, 20 MD, 28 controls	Click and 500 Hz TB (AC) (cVEMP and oVEMP)	Amplitude	VM and MD showed reduced click-cVEMP/oVEMP amplitudes; TB-oVEMP differentiated MD from VM.
Taylor et al., 2012 [31]	60 VM, 60 MD, 30 controls	Click + 250–2000 Hz TB (AC and BC) (oVEMP and cVEMP)	Amplitude, latency	No VEMP differences between VM and controls; MD showed more abnormalities.
Islam et al., 2023 [32]	31 VM	500 Hz TB (cVEMP and oVEMP)	Latency, amplitude, AR	No significant abnormalities in VEMP. oVEMP was more affected than cVEMP.
Murofushi et al., 2009 [33]	11 VM, 11 MD, 8 controls	250–2000 Hz TB	Amplitude, latency	27% showed abnormal frequency tuning (1000-Hz dominance); some prolonged latencies.
Dispenza et al., 2021 [34]	30 VM	500 Hz TB and clicks (cVEMP and oVEMP)	Latency, amplitude, electrocochleography, vHIT	High AR in VM.
Sun et al., 2017 [35]	30 VM, 30 MD	500 Hz TB (threshold testing) (cVEMP and oVEMP)	Threshold, latency, amplitude	No difference in latency amplitude across groups.
Elmoazen et al., 2020 [36]	10 VM, 10 migraine, 10 CG	500 Hz TB (cVEMP and oVEMP)	Latency, amplitude, IAD	VM showed prolonged P13 latency; oVEMP was normal.
Sanitha and Sinha et al., 2024 [37]	30 VM, 30 CG	500 Hz TB	Latency, amplitude, AR	VM had prolonged latencies and reduced amplitudes.
Swain et al., 2020 [38]	51 VM	Not specified (cVEMP and oVEMP)	c/oVEMP presence, caloric test, VNG	63% abnormal cVEMP, 75% abnormal oVEMP.
Khalil et al., 2016 [39]	20 VM, 20 controls	500 Hz TB (cVEMP and oVEMP)	Latency, amplitude, AR	oVEMP was more frequently abnormal (95%); cVEMP abnormalities in 75%.
Utkur et al., 2013 [40]	26 VM, 26 MD, 22 migraine, 27 CG	500 Hz TB	Threshold, amplitude, AR	VM had higher thresholds and reduced amplitudes; MD did not differ significantly from VM.
Sanyelbhaa Talaat and Sanyelbhaa Talaat et al., 2014 [41]	50 VM, 60 controls	500 Hz TB (cVEMP and oVEMP)	Amplitude	VM showed high prevalence of abnormal c/oVEMP and caloric results.
Nafie et al., 2011 [42]	55 VM	512 Hz TB	Presence, latency	VEMP present in 66%, absent in 34%; latencies normal.
Tang et al., 2025 [43]	49 MD, 32 VM, 27 CG	500 Hz, 750 Hz, 1000 Hz TB (cVEMP and oVEMP)	Amplitude, frequency tuning, frequency–amplitude ratio, vHIT, caloric test	cVEMP amplitude decreased at higher frequencies, and oVEMP increased.
Anand and Sarda, 2025 [44]	43 CG, 22 VM, 21 MD	500 Hz, 750 Hz, 1000 Hz, 2000 Hz TB (cVEMP)	Latency, amplitude, and Inter-Frequency Amplitude Ratio	Prolonged P1 latency at 500 Hz, reduced amplitude at all frequencies in MD compared to VM, and higher AFAR at 1000/500 Hz in MD.

Note: VM—vestibular migraine, AC—air conducted, TB—tone bursts, FR = frequency ratio, AR = asymmetry ratio, SCM = sternocleidomastoid, vHIT—video head impulse test, VNG—videonystagmography, cVEMP—cervical vestibular evoked myogenic potential and ocular vestibular evoked myogenic potential, MD—Meniere’s disease, fHIT—functional head impulse test, PTA—pure tone audiometry, SVV—subjective visual vertical, DHI—Dizziness Handicap Inventory, BC—bone conducted.

## Data Availability

No new data were created or analyzed in this study. Data sharing is not applicable to this article.

## Abbreviations

The following abbreviations are used in this manuscript:

VEMP	Vestibular evoked myogenic potential.
VM	Vestibular migraine.
MD	Meniere’s disease
PRISMA	Preferred Reporting Items for Systematic Reviews and Meta-Analyses
PTA	Pure tone audiometry
VNG	Videonystagmography
vHIT	Video head impulse test
AR	Asymmetry ratio
